# Accelerated Ovarian Aging Among Type 2 Diabetes Patients and Its Association With Adverse Lipid Profile

**DOI:** 10.3389/fendo.2022.780979

**Published:** 2022-03-30

**Authors:** Yahao Wang, Yangang Wang

**Affiliations:** ^1^Affiliated Hospital of Qingdao University, Qingdao, China; ^2^Huashan Hospital, Fudan University, Shanghai, China

**Keywords:** ovarian aging, type 2 diabetes mellitus, luteinizing hormone, follicle stimulating hormone, lipids

## Abstract

**Background:**

The impact of diabetes on reproductive function is still not clearly defined. This study aimed to evaluate accelerated ovarian aging in women with type 2 diabetes mellitus (T2DM) and its association with adverse lipid profile.

**Methods:**

Female patients with T2DM (n=964) and non-T2DM controls (n=263) aging from 18-80 years were included. Levels of circulating sex hormones were measured at the follicular phase in menstruating women. We analyzed the age-specific trends in the levels of sex hormones between T2DM and controls. The correlations of sex hormones with the lipid profile, including low-density lipoprotein cholesterol (LDL-C), high-density lipoprotein cholesterol (HDL-C), total cholesterol (TC) and triglycerides (TG) were also evaluated.

**Results:**

In the temporal trends analysis, LH and FSH both started to increase obviously approximately from the age of 45 years among patients with T2DM, and displayed peaks of LH and FSH among patients with T2DM aged between 61 and 65, both of which were obviously earlier than that in non-T2DM controls and proved the accelerated ovarian aging among patients with T2DM. E2 of patients with T2DM was continuous lower than that of non-T2DM controls from approximately 45 years old, and LH and FSH of patients with T2DM were higher than those of non-T2DM controls between the age of 55 to 65 years. Multiple linear regression analyses revealed that LH was positively correlated with LDL-C (Coefficient=0.156, P=0.001) and TC (Coefficient=0.134, P=0.025), and was negatively correlated with HDL-C (Coefficient =-0.065, P=0.001) in patients with T2DM aged between 51 and 60, which was independent of age, T2DM duration, body mass index (BMI), glycosylated hemoglobin (HbA1c), FSH, E2 and other potential confounders. Higher E2 level was significantly and independently correlated with lower LDL-C (Coefficient= -0.064, P=0.033) in patients with T2DM aged between 51 and 60.

**Conclusions:**

This study suggests that patients with T2DM have accelerated ovarian aging, and it is correlated with the occurrence of disturbed lipid profile in patients with T2DM. With an ever increasing number of female patients with T2DM diagnosed at younger ages, the accelerated ovarian aging and its adverse impacts in T2DM need to be carefully managed.

## Introduction

Type 2 diabetes mellitus (T2DM) is a common disease with a serious global pandemic, and could cause substantial adverse impacts on human health (1). The dramatically rising global prevalence of T2DM especially in young adults further increases its global burden (2). Apart from those common diabetic complications such as diabetic neuropathy and diabetic retinopathy, reproductive dysfunction or complications such as subfertility are also prevalent in diabetic patients (3). Ovarian aging commonly refers to the decline of female ovarian functions with age, accompanied by the deterioration in the quantity and quality of ovarian follicles, and dysfunction of granulosa cells and theca cells. (4, 5) Some previous studies have found an association between type 1 diabetes mellitus and accelerated ovarian aging (6–8), and a few studies based on animal models demonstrate that diabetes-obesity syndrome may be associated with impaired oocyte quality (9, 10). However, clinical research on the association between T2DM and accelerated ovarian aging is limited, and the results have been inconsistent. Some studies reported accelerated ovarian aging in patients with T2DM (11–13) while others found no relationships between T2DM and reproductive aging (8, 14).

Cardiovascular complications and microvascular complications are common in patients with T2DM (15, 16). Cardiovascular disease (CVD) is the leading cause of mortality in postmenopausal women (17, 18), and the morbidity rate of CVDs in DM patients is more than twice of that of non-diabetic patients (19). As one of the major causes of atherosclerotic cardiovascular diseases, the dysregulation of lipid metabolism serves as a common feature of T2DM, traditionally characterized by elevated fasting and postprandial triglycerides (TC), low high density lipoprotein-cholesterol (HDL-C), high low density lipoprotein-cholesterol (LDL-C) (20–22). As one of the major characteristics of dyslipdemia, increased LDL-C is an independent risk factor for the development of CVDs, which in women usually occurs in the postmenopausal state (23–25). Among sex hormones, negative associations of E2 with TC and LDL-C have been demonstrated in estrogen receptor (ERα) -deficient models, postmenopausal women with E2 deficiency and women on E2 treatment (26–28). However, studies on the relations between gonadotropins– luteinizing hormone (LH) and follicle-stimulating hormone (FSH)– and lipid levels are still limited and have reported inconsistent findings (27, 29–31). For instance, emerging evidence indicates that FSH level may relate to CVD risks through its association with lipid levels, and some observational studies on women in the menopausal transition demonstrates the association positive (32–35), while other studies on postmenopausal women and older postmenopausal women show it negative (18, 30, 31). Furthermore, clinical investigations show that adoption of hormone replacement therapy (HRT) based on E2 level alone only partially reversed dyslipidemia or lipid oxidation in postmenopausal women. These lead us to hypothesize that gonadotropins may have independent impacts on the lipid profile in perimenopausal or postmenopausal women with T2DM.

The primary purpose of this study is to assess the differences in the age-specific trends of sex hormones in female patients with T2DM and non-T2DM controls, thereby evaluating accelerated ovarian aging in women with T2DM; we also aim to study the correlations of sex hormones with the lipid profile among patients with T2DM.

## Methods

### Study Population

**Figure 1:**
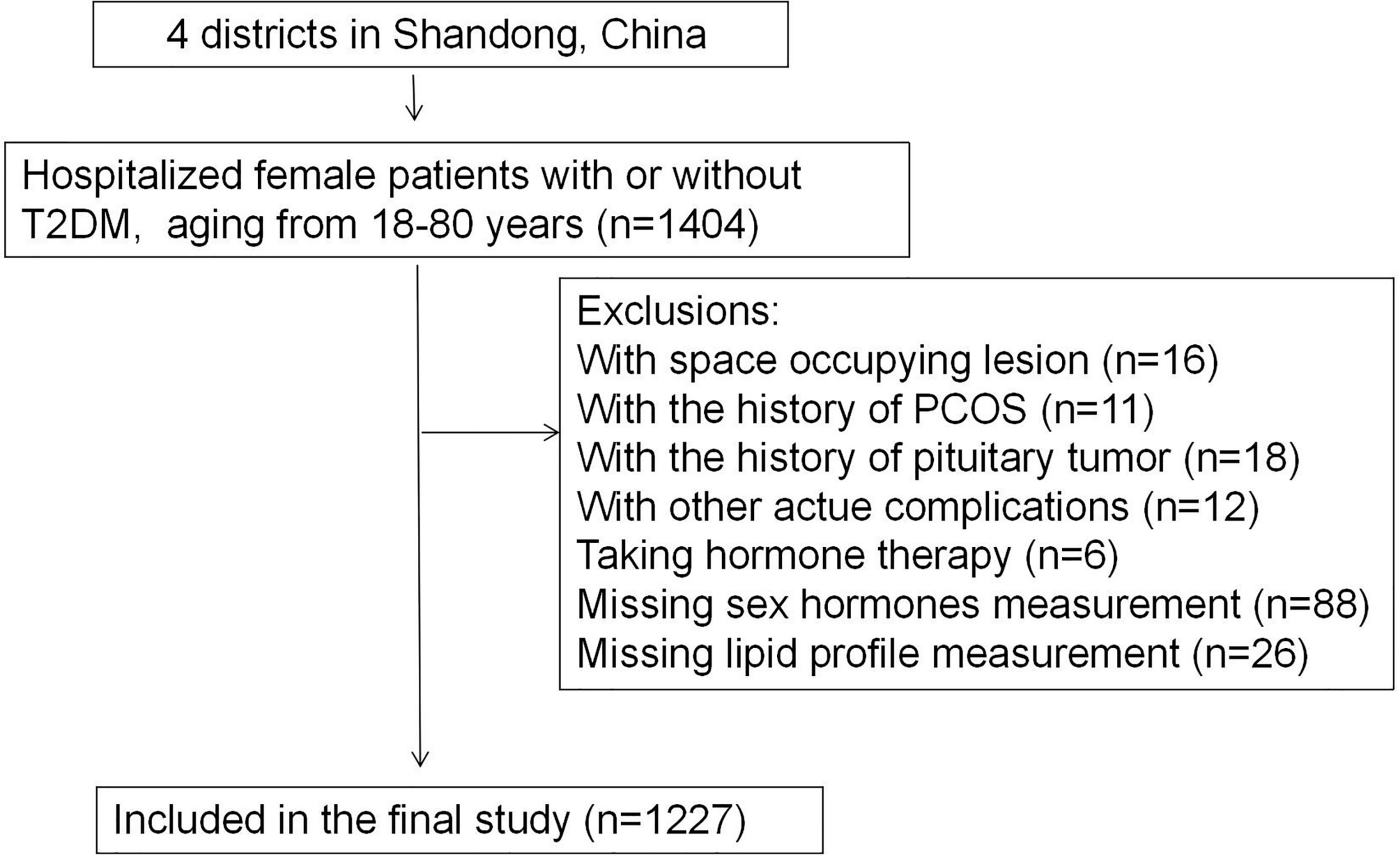
Flowchart of the inclusion and exclusion of participants (T2DM, type 2 diabetes mellitus; PCOS, polycystic ovarian syndrome).

Participants were selected from the database of inpatients at Affiliated Hospital of Medical College Qingdao University (Shandong, China). The inclusion criteria were: female patients with or without T2DM aging between 18 and 80, hospitalized between 2013 and 2020. The exclusion criteria were: patients with space occupying lesion, history of polycystic ovarian syndrome or pituitary tumor, acute complications of T2DM, autoimmune diease and who reported taking hormone therapy, as is shown in **Figure 1**. Thus, we included 964 female patients diagnosed with T2DM and 263 non-T2DM controls in the final study. The protocol was designed according to the Declaration of Helsinki and was approved by the ethics committee of the Affiliated Hospital of Qingdao University. The study was registered on http://www.chictr.org.cn/under number ChiCTR2100045896.

### Anthropometric and Laboratory Data

The characteristics of the study population include age, sex, height, weight and hip circumference, smoking and drinking status, diabetes duration, blood glucose, blood pressure (BP), lipid profiles including LDL-C, HDL-C, TC, triglycerides (TG), free fatty acid (FFA), liver and renal function, and personal medical history. Clinical examinations were conducted by a trained staff group according to a standard guideline. In particular, height and weight were measured with patients standing without shoes and with lightweight clothing. Waist circumference was measured at the midpoint of the lowest rib and the iliac crest. BP was reported as the means of three consecutive measurements with an interval of 5 minutes.

Blood samples were obtained between 6:00 and 9:00 AM after fasting for at least 8 hours. Venipuncture was performed in the median cubital vein, and centrifugation and dispensing of serum separated from blood samples were completed within 1 hour. Levels of circulating sex hormones were measured at the follicular phase in menstruating women. All samples were transported under cold chain to a central laboratory for testing within 2 to 4 hours. Sex hormones including total testosterone (TT), estradiol (E2), FSH, and LH were measured by electrochemiluminescence immunoassay (Roche E602, Roche, Basel, Switzerland). The minimal detectable limits for each hormone were as follows: 0.09 nmol/L (TT), 18.35 pmol/L (E2), and 0.1 IU/L (LH and FSH). Glycosylated hemoglobin (HbA1c) was assessed by high-performance liquid chromatography (MQ-2000PT, Medconn, Shanghai, China). Serum glucose and lipid profiles were measured by Beckman Coulter AU 680 (Beckman Coulter, Krefeld, Germany). Body mass index (BMI) was calculated as weight divided by height squared (kg/m^2^).

### Statistical Analysis

The SPSS version 22.0 software and R were used to perform statistical analyses. Continuous variables are presented as median (interquartile range); categorical variables are presented as frequency (%). Mann-Whitney test was used for testing differences between T2DM group and non-T2DM control group for continuous variables, Kruskal-Wallis H test for testing differences between age groups and chi-square test for categorical variables. To assess the temporal trends in circulating sex hormones across age groups, curves were fitted using three models (linear model, Locally Weighted regression (LOESS) model and binary linear regression model), and R^2^ representing the coefficient of determination in the linear model was calculated. The correlations between the levels of sex hormones and lipid profile variables were further assessed by multiple linear regression and results were expressed as standardized coefficients, in which potential confounders such as age, T2DM duration, BMI, HbA1c and blood pressures were adjusted. LH, FSH and E2 levels were divided into four quartiles, with the first quartile (Q1) representing the lowest quartile and the fourth quartile (Q4) being the highest quartile. All statistical analyses were two sided; and P values less than 0.05 were considered to be statistically significant.

## Results

### Patients’ Characteristics and the Changes of Circulating Sex Hormones Across Age

General demographic and laboratory characteristics of the participants were summarized in **Supplemental Table 1** and **Supplemental Table 2**. This study recruited 964 female patients with T2DM and 263 non-T2DM controls. The median age of patients with T2DM and non-T2DM controls were respectively 62 and 59 years, and percentage of menopause were respectively 87.1% and 73.04%; patients with T2DM were more likely to experience higher BMI compared with controls (**Table 1**). Compared with non-T2DM controls, patients with T2DM had significantly lower levels of LH (P<0.001), FSH (P<0.001) and E2 (P<0.001); no difference in the testosterone level between patients with T2DM and non-T2DM controls was observed (P=0.421; **Table 1**). The decreased levels of LH, FSH and E2 in patients with T2DM are consistent with findings from previous literatures (36–39), which may be partially attributed to the impairment of hyperglycemia on hypothalamic function (40–42).

**Table 1:** Summary of clinical features of patients with T2DM and non-T2DM controls.

Items	T2DM	Non-T2DM controls	P^a^
Number	964	263	
Age, year	62 (55, 69)	59 (51, 67)	0.001
DM duration, year	10 (5, 17)	–^*^	–^*^
BMI, kg/m^2^	25.6 (23.3, 28.0)	24.1 (22.1, 26.7)	<0.001
Menopause (%)	840 (87.1%)	192 (73.04%)	<0.001
LDL-C, mmol/L	2.73 (2.16, 3.38)	2.83 (2.22, 3.46)	0.224
HDL-C, mmol/L	1.26 (1.07, 1.48)	1.50 (1.31, 1.74)	<0.001
TG, mmol/L	1.42 (0.99, 2.16)	1.05 (0.73, 1.54)	<0.001
TC, mmol/L	4.62 (3.91, 5.45)	4.96 (4.20, 5.78)	<0.001
LH, mIU/ml	21.75 (14.35, 29.79)	26.47 (17.77, 35.10)	<0.001
FSH, mIU/ml	45.51 (29.51, 60.65)	53.39 (35.44, 70.35)	<0.001
T, nmol/L	0.55 (0.28, 0.88)	0.58 (0.35-0.84)	0.421
E2, pmol/L	30.84 (18.35, 72.23)	64.28 (37.94-144.30)	<0.001

Data were shown as median (interquartile range). BMI, body mass index; DM, diabetes mellitus; LDL-C, low-density lipoprotein cholesterol; HDL-C, high-density lipoprotein cholesterol; TG, triglyceride; TC, total cholesterol; LH, luteinizing hormone; FSH, follicle-stimulating hormone; T, testosterone; E2, estradiol. ^a^Mann-Whitney test or chi-square test. ^*^Not applicable.

In general, patients with T2DM in older age groups were more likely to experience longer diabetes duration, higher systolic blood pressure and lower diastolic blood pressure (P<0.001); patients in the eldest group were likely to experience lower LDL-C and TC levels, where the increased percentage of statins as medication may play a role; differences of TG and FFA between age groups didn’t reach statistical significance (**Supplemental Table 2**). LH and FSH levels were significantly higher in groups more than 51 years old, while the T and E2 showed an opposite trend.

### Patients With T2DM Had Accelerated Ovarian Aging

**Figure 2:**
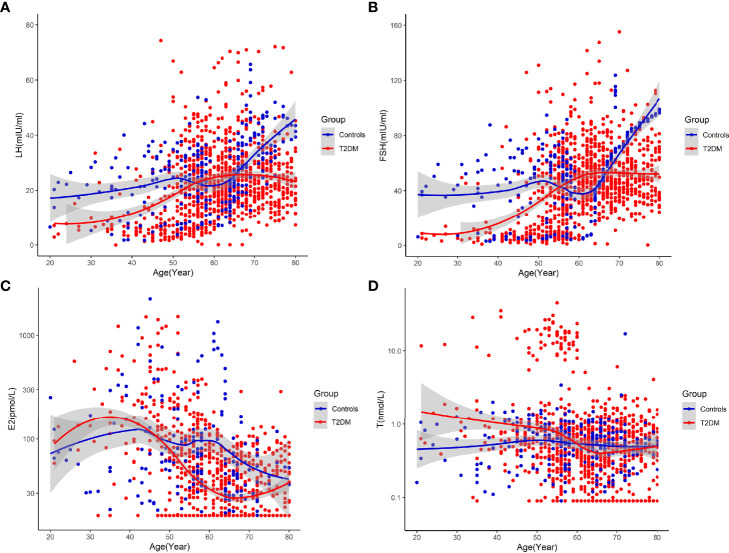
Temporal trends in circulating sex hormones across age groups in patients with T2DM and non-T2DM controls [**(A)** Temporal trends in circulating LH across age groups. **(B)** Temporal trends in circulating FSH across age groups. **(C)** Temporal trends in circulating E2 across age groups. **(D)** Temporal trends in circulating testosterone (T) across age groups].

In the temporal trends analysis,we got the following results: (1) LH and FSH both started to increase obviously approximately from the age of 45 years among patients with T2DM, and displayed peaks of LH and FSH among patients with T2DM aged between 61 and 65, both of which were obviously earlier than that in non-T2DM controls (**Figures 2A, B**). E2 started to decrease obviously from earlier than 40 years old among patients with T2DM, while it started to decrease obviously approximately from the age of 45 years among non-T2DM controls (**Figure 2C**). The earlier changes in sex hormones suggests accelerated ovarian aging in patients with T2DM. (2) E2 of patients with T2DM was continuous lower than that of non-T2DM controls from approximately 45 years old, thus proving accelerated ovarian aging in patients with T2DM. On the other hand, LH and FSH of patients with T2DM were higher than those of non-T2DM controls between the age of 55 and 65 years. The rise in levels of LH and FSH after the decrease in E2 level could be due to the slightly delayed feedback of aggravated E2 deficiency in the secretion of gonadotropins.

**Figure 3:**
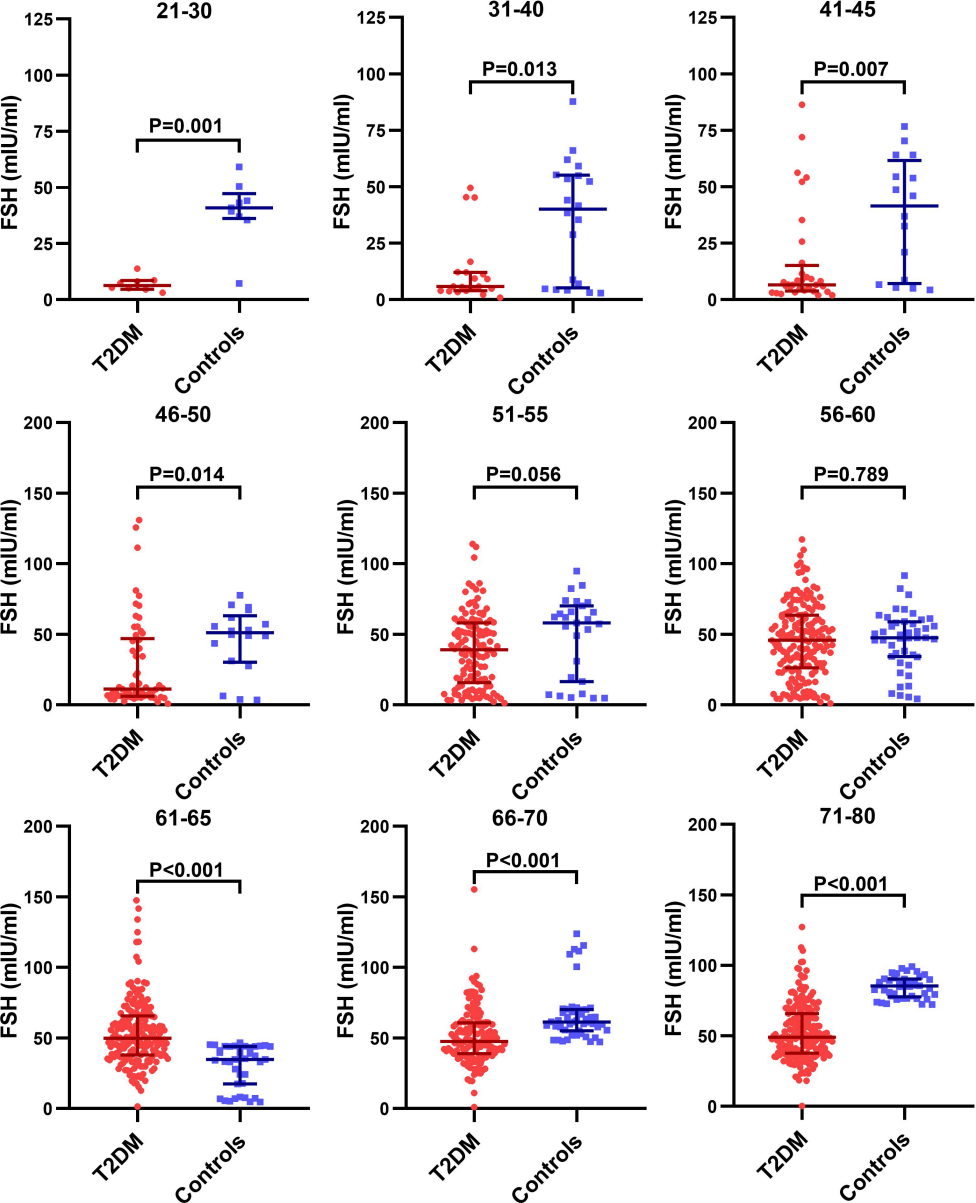
Difference in the circulating FSH level between patients with T2DM and non-T2DM controls stratified by age.

**Figure 4:**
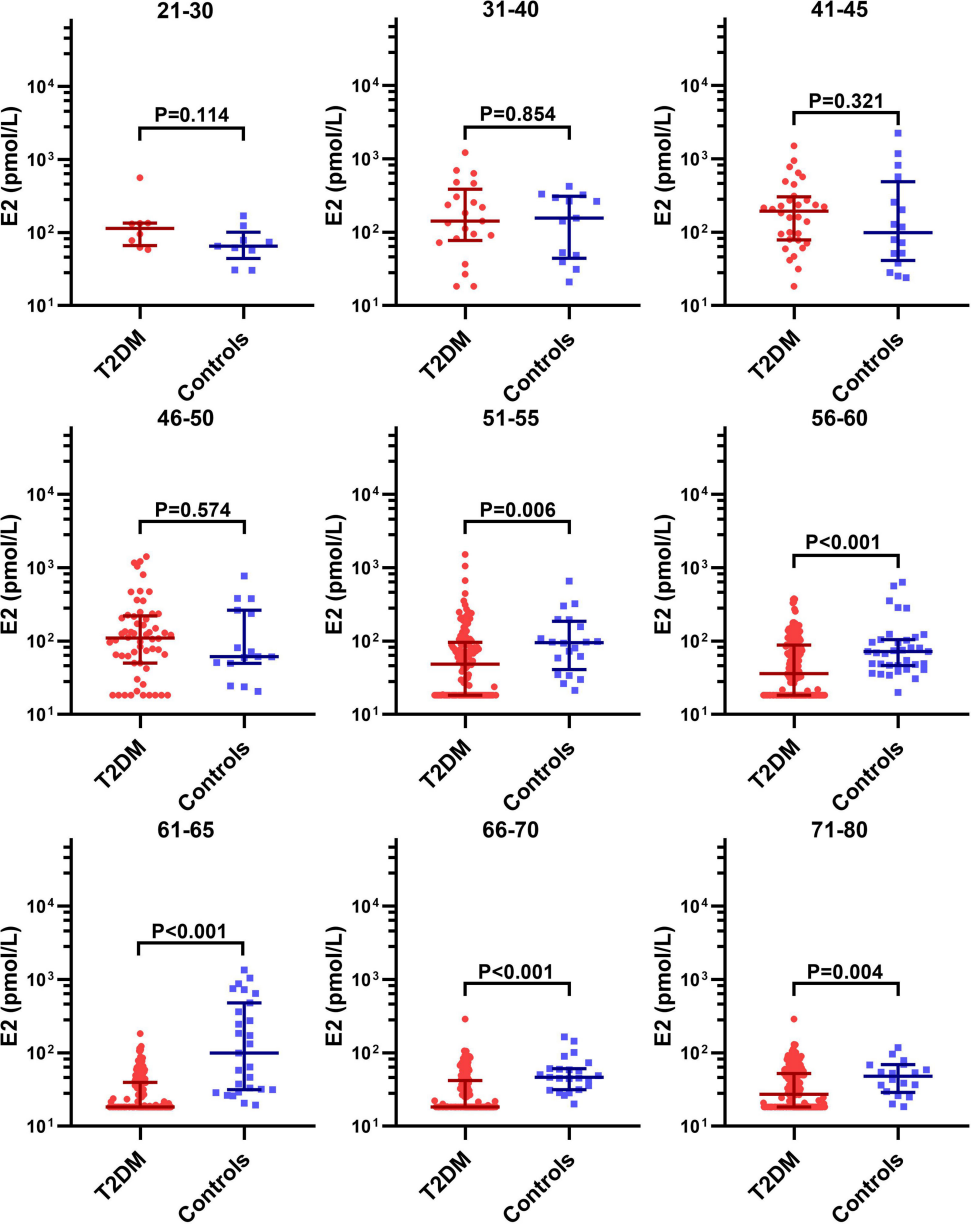
Difference in the circulating E2 level between patients with T2DM and non-T2DM controls stratified by age.

We further analyzed the age-specific trends in sex hormones by clarifying differences in the circulating sex hormones between patients with T2DM and non-T2DM controls stratified by age. Patients with T2DM had significantly lower LH and FSH than non-T2DM controls before 50 years old, but the difference disappeared in the age groups of 51-60 years (**Supplemental Figure 1** and **Figure 3**). Both LH and FSH were significantly higher in patients with T2DM compared with non-T2DM controls in the age groups of 61-65 years (p < 0.001). Moreover, patients with T2DM appeared to keep having significantly lower E2 level than non-T2DM controls from the age of 51 years (**Figure 4**). The findings above confirmed the age-specific differences in sex hormones between women with and without T2DM indicated by **Figure 2**.

### Correlations of Sex Hormones With Lipid Profile in Patients With T2DM Aged Between 51 and 60

As **Figure 3**, **Supplemental Figure 1** and **Figure 4** suggests, the aging-related changes in sex hormones of patients with T2DM were much more obvious in the age group of 51-60 years than the earlier age groups. Given that our second aim is to evaluate the association of accelerated ovarian aging with adverse lipid profile including LDL-C, HDL-C, TC, TG and FFA in patients with T2DM, we chose the age group of 51-60 years to assess the relationship of sex hormones with lipid profile considering that: (1) levels of sex hormones in this age group not only show drastic changes, but also were approximate to perimenopausal levels, thus worth investigating; (2) this age group was also characterized by disturbed lipid profile (**Supplemental Table 2**).

In the analyses of clinical characteristics of patients with T2DM aged between 51 and 60 by the quartiles of LH and FSH (**Supplemental Tables 3**, **4**), patients with the higher level of LH and FSH were more likely to experience significantly higher TC and HDL-C compared with those with the bottom quartile of LH and FSH.

**Figure 5:**
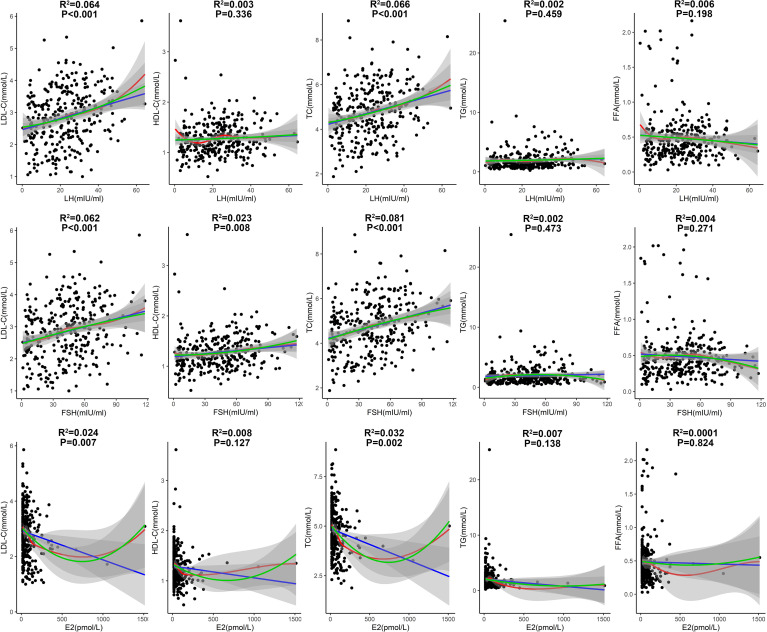
Correlations of lipid profile with LH, FSH and E2 in patients with T2DM aged 51-60 years. (Fitted curves of linear model are shown in blue; smoothing fitted curves of LOESS model are in red; fitted curves of binary linear regression model are in green. Adjusted R^2^ and P values from the linear model are shown in the figure).

The correlations of lipid profile with LH, FSH and E2 in patients with T2DM aged 51-60 years were then analyzed (**Figure 5**). LH had significantly positive correlations with LDL-C and TC levels (P<0.001; **Figure 5**), suggesting a driving role of LH in dyslipidemia. FSH had significant positive correlations with LDL-C and TC levels (P<0.001), and had a modest positive correlation with HDL-C (P=0.008; **Figure 5**). E2 had significantly negative correlations with LDL-C and TC levels (P<0.01; **Figure 5**), suggesting a protective role of E2 against dyslipidemia.

In the multiple linear regression analyses by adjusting for age, T2DM duration, BMI, blood pressures, HbA1c, sex hormones and other potential confounders (**Table 2**), LH was positively correlated with LDL-C (Coefficient=0.156, P=0.001) and TC (Coefficient=0.134, P=0.025), and was negatively correlated with HDL-C (Coefficient =-0.065, P=0.001) in patients with T2DM aged between 51 and 60. Higher E2 level was significantly and independently correlated with lower LDL-C (Coefficient= -0.064, P=0.033) in patients with T2DM aged between 51 and 60.

**Table 2:** Correlations of lipid profile with LH or FSH in patients with T2DM aged 51-60 years.

Variable	LH (mIU/mL)		FSH (mIU/mL)		E2 (pmol/L)	
	Coefficient^*^	P value	Coefficient^#^	P value	Coefficient^&^	P value
LDL-C (mmol/L)	0.156	0.001	-0.079	0.127	-0.064	0.033
HDL-C (mmol/L)	-0.065	0.001	0.078	<0.001	-0.011	0.373
TG (mmol/L)	0.027	0.809	-0.068	0.584	0.049	0.502
TC (mmol/L)	0.134	0.025	0.0001	0.999	-0.046	0.240
FFA (mmol/L)	-0.018	0.402	-0.006	0.782	0.004	0.782

The values of LH, FSH, E2 and testosterone were log2-transformed, and multiple linear regression analyses were performed. *Adjusted for age, T2DM duration, BMI, blood pressure, smoking, drinking, HbA1c, FPG, uric acid, FSH, testosterone and E2. ^#^Adjusted for age, T2DM duration, BMI, blood pressure, smoking, drinking, HbA1c, FPG, uric acid, LH, testosterone and E2. ^&^Adjusted for age, T2DM duration, BMI, blood pressure, smoking, drinking, HbA1c, FPG, uric acid, FSH, LH and testosterone. LDL-C, low-density lipoprotein cholesterol; HDL-C, high-density lipoprotein cholesterol; TG,triglyceride; TC, total cholesterol; FFA, free fatty acid.

## Discussion

A major finding in this study is the accelerated ovarian aging among patients with T2DM. Reproductive aging such as premature ovarian failure can promote the development of cardiovascular diseases and has been reported among patients with T1DM (6–8). However, the reproductive aging of patients with T2DM is less well understood, and evidence from clinical studies is still limited. In the present study, we found that LH and FSH both started to increase obviously approximately from the age of 45 years among patients with T2DM, and displayed peaks of LH and FSH among patients with T2DM aged between 61 and 65, both of which were obviously earlier than that in non-T2DM controls; meanwhile, E2 started to decrease obviously approximately from the age of 40 years among patients with T2DM, while it started to decrease obviously approximately from the age of 45 years among non-T2DM controls (**Figure 2**). The findings above prove that aging-related changes in sex hormones occur earlier in patients with T2DM and patients with T2DM have accelerated ovarian aging than controls.

The age-specific trends of LH, FSH and E2 in patients with T2DM and non-diabetic controls are consistent with findings from several studies (11–13). Previous clinical research found a decline in ovarian volume, total antral follicle count, inhibin B secretion and anti-Miller hormone level in patients with T2DM compared with healthy controls, all of which were also markers for ovarian aging (9, 43). And a few studies based on animal models demonstrated that diabetes-obesity syndrome was associated with suppression of cyclic ovarian follicular recruitment patterns, anovulation, acyclicity, depressed ovarian steroid hormone synthesis and release, hypovascularization and tissue ischemia, enhanced follicular atresia, premature tissue atrophy and involution (9, 10). Despite such supporting evidence for accelerated ovarian aging in patients with T2DM, the underlying mechanisms are far from being clarified. Here, we deduced several possible mechanisms as follows: 1) It’s well established that low-grade chronic inflammation is involved in the pathophysiology T2DM (44), due mainly to the activation of the myeloid cell lineage (e.g., macrophages and neutrophils) (45). Inflammation of the whole body can affect oocytes *via* the follicle microenvironment and oxidative stress (46), thus impairing oocyte meiosis and oocyte quality (47). Indeed, chronic inflammation is found in aging ovaries, which is proved by the increase of multiple proinflammatory transcription factors, cytokines and chemokines (4, 48). For example, TNF-α binds to its death receptors (such as Fas) and then activates apoptosis of granular cells and oocytes through Fas-associated death domain-dependent activation of caspase-8; while TNFα knockout mice proved higher levels of granular cell proliferation (49). In addition, macrophage infiltration could induce the accumulation of collagen fibers in extracellular matrix, thereby promoting ovarian fibrosis (50). 2) Oxidative stress plays a key role in the pathophysiology of T2DM. During the progression of diabetes, hyperglycemia promotes mitochondrial dysfunction and induces the formation of oxygen species (ROS) that causes oxidative stress in several tissues including ovaries (45). And several studies have shown that the accumulation of ROS in the ovaries deteriorates oocyte quality, induces granulosa cell apoptosis, and accelerates degeneration of the corpus luteum (51). Excessive ROS may change the mitochondrial membrane potential by regulating the ratio of pro-/anti-apoptosis factors (49, 52), which eventually leads to the activation of caspase-3 which cleaves various structural and regulatory proteins in ovarian cells and causes apoptosis (46, 49). Moreover, oxidative stress and the accumulation of acquired mtDNA mutations could cause mtDNA damage (53). The effect of mtDNA alterations in ovarian follicle cells on qualitative ovarian reserve depletion and ovarian aging is also supported by the robust evidence (46, 53), though the mechanisms require further probe. 3) Vascular damage is one of the most common complications of diabetes, characterized by endothelium cell dysfunction, impaired angiogenesis, platelet activation and subsequent microembolisms (45). According to previous research, vascular impairment could lead to early menopause (54), in which the pathophysiological features of diabetic vascular damage may play an important part. And it’s worth noticing that dyslipidemia also could accelerate the impairment of oocyte quality, as it promotes endothelial dysfunction and platelet hyperactivation, potentially causing vascular damage and imposing cytolipotoxic effects (9, 45). 4) Diabetes is known to be associated with an accelerated rate of the accumulation of advanced glycation end products (AGEs) (55). The accumulation of AGEs has been reported to be closely related to declining ovarian function (56). The glycation process causes protein damage, and the interaction of AGEs and their receptors could lead to the activation of a number of proinflammatory pathways, increased production of ROS in oocytes, and endothelial dysfunction *via* inactivation of endothelial nitric oxide synthase and prostacyclin synthase, thereby inducing a premature decline in ovarian function (48, 56). Moreover, AGEs were reported to accelerate the progression of nuclear maturation, though the mechanisms still unknown (57).

We also found that LDL-C and TC levels were positively correlated with LH and FSH, and negatively correlated E2 with LDL-C and TC levels in patients with T2DM aged between 51 and 60, suggesting perimenopausal changes of gonadotropins and E2 are correlated with dyslipidemia. According to previous studies, consistent cross-sectional and longitudinal associations of menopause with worsening lipid profile have been observed (58), and for decades the associations have been mainly attributed to estrogen deficiency. Negative associations of E2 with TC and LDL-C has been demonstrated in ERα-deficient models, women with E2 deficiency and postmenopausal women on E2 treatment (59–65), results consistent with our study findings. These protective effects of E2 can be largely attributed to its upregulation of low-density lipoprotein receptors (LDLR) on hepatic cells: 1) through nuclear receptor ER, E2 stimulates the expression of transcription factors related to cholesterol metabolism, which upregulates LDLR expression as a downstream target (66); 2) through G protein-coupled estrogen receptor, E2 prevents PCSK9-dependent LDLR degradation, thus reducing the levels of TC and LDL-C as well (67). In terms of gonadotropins, although studies regarding the role of LH in dyslipidemia are scarce, several previous studies addressing the links between FSH and lipid levels in postmenopausal women have demonstrated that higher FSH in postmenopausal women was related to higher levels of both TC and LDL-C, with stronger relations seen in younger compared to older postmenopausal women, which were close to the results of our results (29, 68). In the animal model of ovariectomized mice which simulated the postmenopausal period, an increase in both TC and LDL-C was also found when FSH was elevated independent of E2 (68); in postmenopausal women, adjusting the estrogen dose in HRT to attain a target FSH decrease might lead to beneficial alterations in TC and LDL-C (68). Further, studies on women in the menopausal transition suggests that an increase in FSH accompanying the menopause transition may contribute to the higher risk of CVDs including subclinical atherosclerosis after menopause (32–34). The biological mechanisms identified so far come down to the downregulation of LDLR expression (68). LDLRs in the liver determine the turnover rate of LDL-C and thus regulate the plasma LDL concentrations. FSH may interact with its receptors (FSHR) in hepatocytes and reduce LDLR levels, which subsequently attenuates the endocytosis of LDL-C, resulting in an elevated circulating LDL-C level (68). All in all, FSH might reduce the uptake and metabolism, especially degradation of LDL-C in liver (68).

According to our study, the LH and FSH levels began to increase obviously approximately from 45 years old, indicating the beginning of perimenopausal stage, which commonly lasts 2 to 10 years and is characterized by less regular menstruation (69); the relations between gonadotropins and LDL-C and TC were found in female patients with T2DM aged between 51 and 60, generally a perimenopausal or early postmenopausal state. Correspondingly, the indication from 2019 ACC/AHA Guideline on the Primary Prevention of Cardiovascular Disease directly recommends patients 40 to 75 years of age with LDL-C>70 mg/dL (1.8 mmol/L) to start moderate-intensity statin therapy for primary prevention once diagnosed with DM, indicating that the cholesterol management in women with T2DM should start from a relative early age to prevent from CVDs (70). Interestingly, randomized controlled trials suggest that hormone treatment achieves most cardiovascular benefits if initiated soon after menopause, and is potentially harmful if initiated late (>10 years) after menopause (26). However, the early prevention is not fully implemented clinical work, and diabetic women are less likely to be on optimal lipid-lowering therapy compared to men (71), though the British Heart Foundation states that the number of women living with CVDs is now roughly the same as the number of men (26). Combined with our finding of T2DM accelerating ovarian aging, more research is needed on metabolic changes in women with T2DM of an age range linked with drastic changes of sex hormone levels.

This study had some limitations. The retrospective study cannot infer cause and effect; the restricted sample size may not be generalized to the larger population; there is insufficient experimental evidence to explain the relationships we found. Further, the varied amount of subjects taking statins between age groups and groups divided by LH and FSH quartiles could cause bias in results; but the correlations we found in subjects aged between 51 and 60 were more likely to be underestimated since the percentage of statins taking was higher in groups of higher LH and FSH levels compared with groups with the bottom quartile of LH and FSH. Finally, this study emphasizes the necessity of an early management of ovarian aging in patients with T2DM, and lends credibility to the suggestion to adopt the cholesterol management as early as the age of 40 in women with T2DM; while the mechanisms of the association between T2DM and accelerated ovarian aging, and the correlations of gonadotropins with the lipid profile and other cardiovascular risks still require investigation.

Overall, the study indicates that patients with T2DM have accelerated ovarian aging, and the elevated LH and FSH levels together with the decreased E2 level caused by the accelerated ovarian aging are correlated with the occurrence of disturbed lipid profile in patients with T2DM. With an ever increasing number of female patients with T2DM diagnosed at younger ages, the accelerated ovarian aging and its adverse impacts in T2DM need to be carefully managed. Besides, further evaluation of these relations and the mechanisms underlying them is warranted, especially *via* prospective studies that can provide a clearer understanding for accelerated ovarian aging in patients with T2DM.

## Data Availability Statement

The original contributions presented in the study are included in the article/**Supplementary Material**. Further inquiries can be directed to the corresponding author.

## Ethics Statement

The studies involving human participants were reviewed and approved by medical ethics committee of the Affiliated Hospital of Qingdao University. The patients/participants provided their written informed consent to participate in this study. Written informed consent was obtained from the individual(s) for the publication of any potentially identifiable images or data included in this article.

## Author Contributions

YHW: designing and registering the work; the acquisition, analysis or interpretation of data for the work; drafting the work. YGW: agree to be accountable for all aspects of the work in ensuring that questions related to the accuracy or integrity of any part of the work are appropriately investigated and resolved. All authors contributed to the article and approved the submitted version.

## Conflict of Interest

The authors declare that the research was conducted in the absence of any commercial or financial relationships that could be construed as a potential conflict of interest.

## Publisher’s Note

All claims expressed in this article are solely those of the authors and do not necessarily represent those of their affiliated organizations, or those of the publisher, the editors and the reviewers. Any product that may be evaluated in this article, or claim that may be made by its manufacturer, is not guaranteed or endorsed by the publisher.

## Supplementary Material

Supplementary Figure 1 Difference in the circulating LH level between patients with T2DM and non-T2DM controls stratified by age.
