# Supplementary figures and images for: Accelerated Ovarian Aging Among Type 2 Diabetes Patients and Its Association With Adverse Lipid Profile
Source: Front Endocrinol (Lausanne). 2022 Mar 30;13:780979. doi: 10.3389/fendo.2022.780979 (PMC9005646; doi:10.3389/fendo.2022.780979)

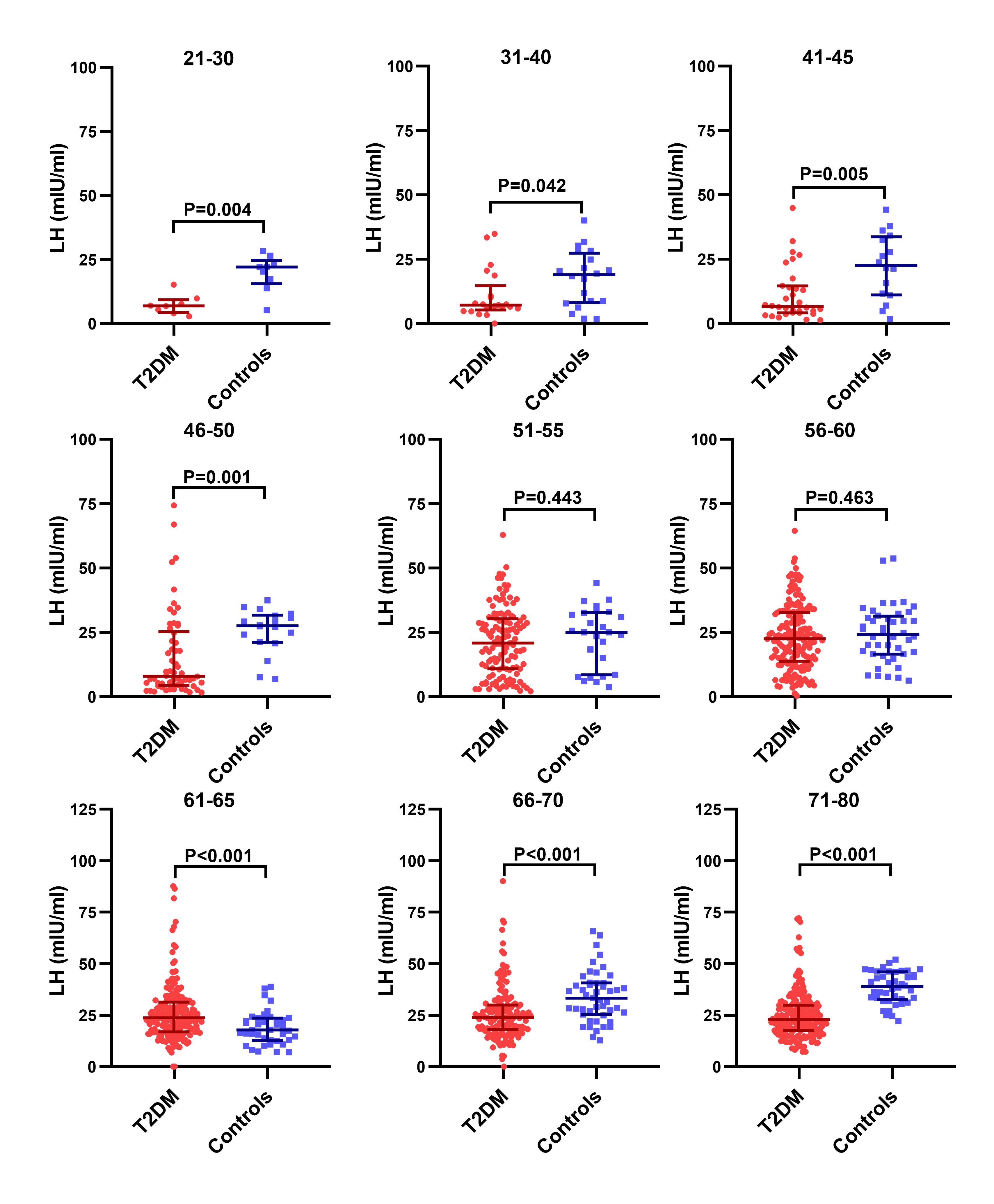

Supplement: Supplementary Figure 1 — Difference in the circulating LH level between patients with T2DM and non-T2DM controls stratified by age. [file Image_1.jpeg]
