# Supplementary material for: Accelerated Ovarian Aging Among Type 2 Diabetes Patients and Its Association With Adverse Lipid Profile
Source: Front Endocrinol (Lausanne). 2022 Mar 30;13:780979. doi: 10.3389/fendo.2022.780979 (PMC9005646; doi:10.3389/fendo.2022.780979)
Supplement: Supplementary file 2 [file Table_1.docx]

**Supplemental table 1 Summary of clinical features of T2DM patients and non-T2DM controls**

| **Items** | **T2DM** | **Non-T2DM controls** | **P^a^** |
| --- | --- | --- | --- |
| Number | 964 | 263 |  |
| Age, year | 62(55, 69) | 59(51, 67) | 0.001 |
| DM duration, year | 10(5, 17) | **--**^*^ | **--**^*^ |
| BMI, kg/m^2^ | 25.6(23.3, 28.0) | 24.1(22.1, 26.7) | <0.001 |
| Menopause (%) | 840(87.1%) | 192(73.04%) | <0.001 |
| LDL-C, mmol/L | 2.73(2.16, 3.38) | 2.83(2.22, 3.46) | 0.224 |
| HDL-C, mmol/L | 1.26(1.07, 1.48) | 1.50(1.31, 1.74) | <0.001 |
| TG, mmol/L | 1.42(0.99, 2.16) | 1.05(0.73, 1.54) | <0.001 |
| TC, mmol/L | 4.62(3.91, 5.45) | 4.96(4.20, 5.78) | <0.001 |
| LH, mIU/ml | 21.75(14.35, 29.79) | 26.47(17.77, 35.10) | <0.001 |
| FSH, mIU/ml | 45.51(29.51, 60.65) | 53.39(35.44, 70.35) | <0.001 |
| T, nmol/L | 0.55(0.28, 0.88) | 0.58(0.35-0.84) | 0.421 |
| E2, pmol/L | 30.84(18.35, 72.23) | 64.28(37.94-144.30) | <0.001 |

(Note: Data were shown as median (interquartile range). Abbreviations: BMI, body mass index; DM, diabetes mellitus; LDL-C, low-density lipoprotein cholesterol; HDL-C, high-density lipoprotein cholesterol; TG, triglyceride; TC, total cholesterol; LH, luteinizing hormone; FSH, follicle-stimulating hormone; T, testosterone; E2, estradiol. ^a^Mann-Whitney test or chi-square test. ^*^Not applicable.)
