# Supplementary material for: Accelerated Ovarian Aging Among Type 2 Diabetes Patients and Its Association With Adverse Lipid Profile
Source: Front Endocrinol (Lausanne). 2022 Mar 30;13:780979. doi: 10.3389/fendo.2022.780979 (PMC9005646; doi:10.3389/fendo.2022.780979)
Supplement: Supplementary file 3 [file Table_2.docx]

**Supplemental table 2 Summary of clinical features of T2DM patients stratified by age**

| Characteristics | Age groups | | | | | | p^a^ |
| --- | --- | --- | --- | --- | --- | --- | --- |
|  | 21~30 (n=8) | 31~40 (n=21) | 41~50 (n=90) | 51~60 (n=302) | 61~70 (n=344) | 71~80 (n=199) |  |
| BMI, kg/m^2^ | 23.9(22.7,28.6) | 26.0(22.0,27.9) | 25.0(22.9,28.6) | 25.9(23.5,28.2) | 25.5(23.4,28.0) | 25.8(23.4,28.4) | 0.674 |
| WHR | 0.95(0.9,1.0) | 0.92(0.9,1.0) | 0.92(0.9,1.0) | 0.93(0.9,1.0) | 0.93(0.9,1.0) | 0.95(0.9,1.0) | 0.005^**^ |
| T2DM duration, year | 0.6(0.1,2.3) | 4.0(2.0,8.0) | 6.0(2.0,10.0) | 9.0(4.0,13.0) | 11.0(7.0,17.0) | 16.0(10.0,20.0) | <0.001^**^ |
| BP, mmHg |  |  |  |  |  |  |  |
| Systolic | 121.5(115.3,133.3) | 132.0(122.0,146.5) | 133.0(118.8,153.3) | 136.0(124.0,151.3) | 141.0(128.3,154.0) | 146.0(133.0,161.0) | <0.001^**^ |
| Diastolic | 81.5(72.8,84.8) | 85.0(76.5,90.5) | 80.0(73.0,89.0) | 78.0(71.0,84.0) | 76.0(69.0,83.0) | 74.0(66.0,81.0) | <0.001^**^ |
| HbA1c, % | 8.9(6.0,12.2) | 9.3(8.3,10.2) | 8.6(7.0,9.6) | 8.1(6.9,9.6) | 7.8(6.8,9.1) | 8.0(6.8,9.3) | 0.104 |
| Lipid profile, mmol/L |  |  |  |  |  |  |  |
| LDL-C | 2.66(1.9,3.1) | 3.36(2.8,3.9) | 2.94(2.3,3.6) | 2.97(2.4,3.6) | 2.71(2.1,3.4) | 2.43(1.9,3.2) | 0.004^**^ |
| HDL-C | 0.92(0.8,1.2) | 1.20(0.9,1.3) | 1.24(1.1,1.4) | 1.28(1.1,1.5) | 1.31(1.1,1.5) | 1.26(1.1,1.5) | 0.004^**^ |
| TG | 2.20(1.0,5.3) | 2.41(1.2,9.6) | 1.54(0.8,2.6) | 1.49(1.1,2.3) | 1.41(1.0,2.1) | 1.36(1.0,1.9) | 0.215 |
| TC | 4.58(3.4,5.0) | 5.71(4.8,5.8) | 4.85(4.1,5.6) | 4.93(4.2,5.7) | 4.61(4.0,5.4) | 4.43(3.7,5.3) | 0.025^*^ |
| FFA | 0.49(0.4,0.7) | 0.40(0.4,0.7) | 0.42(0.3,0.7) | 0.43(0.3,0.6) | 0.42(0.3,0.6) | 0.45(0.3,0.6) | 0.194 |
| Sex Hormones |  |  |  |  |  |  |  |
| LH, mIU/ml | 6.57(3.7,11,1) | 7.46(5.7,10.8) | 6.63(3.6,17.5) | 24.40(17.7,32.7) | 23.72(17.0,30.1) | 22.85(17.3,29.8) | <0.001^**^ |
| FSH, mIU/ml | 5.20(4.2,7.6) | 5.11(3.9,9.1) | 6.84(5.0,13.8) | 50.71(33.3,64.1) | 48.17(38.3,61.8) | 49.03(37.8,64.7) | <0.001^**^ |
| T, nmol/L | 1.03(0.5,1.7) | 0.81(0.5,1.2) | 0.63(0.4,0.9) | 0.47(0.2,0.7) | 0.48(0.2,0.8) | 0.51(0.3,0.8) | <0.001^**^ |
| E2, pmol/L | 132.50(87.4,242.4) | 235.00(94.5,479.2) | 166.00(74.1,362.8) | 27.30(18.4,40.2) | 18.35(18.4,40.2) | 27.26(18.4,52.1) | <0.001^**^ |
| Statins | 0(0.0) | 4(26.7) | 34(54.0) | 151(74.4) | 268(79.3) | 164(84.5) | <0.001^**^ |

(Note: Data are presented as the median (interquartile range) for continuous variables or percentage for categorical variables. Abbreviations: BMI, body mass index; WHR, waist-to-hip ratio; T2DM, Type 2 diabetes mellitus; BP, blood pressure; HbA1c, glycosylated hemoglobin; LDL-C, low-density lipoprotein cholesterol; HDL-C, high-density lipoprotein cholesterol; TG,triglyceride; TC, total cholesterol; FFA, free fatty acid; LH, luteinizing hormone; FSH, follicle-stimulating hormone; T, testosterone; E2, estradiol. ^a^Kruskal-Wallis H test or chi-square test. ^*^ Significant at p<0.05; ^**^ Significant at p<0.01.)
