# Supplementary material for: Accelerated Ovarian Aging Among Type 2 Diabetes Patients and Its Association With Adverse Lipid Profile
Source: Front Endocrinol (Lausanne). 2022 Mar 30;13:780979. doi: 10.3389/fendo.2022.780979 (PMC9005646; doi:10.3389/fendo.2022.780979)
Supplement: Supplementary file 4 [file Table_3.docx]

**Supplemental table 3 Summary of clinical features of T2DM patients aged 51-60 years stratified by LH quartiles**

| Characteristics | LH quartiles | | | | P^a^  |
| --- | --- | --- | --- | --- | --- |
|  | Q1(n=241) | Q2(n=241) | Q3(n=241) | Q4(n=241) |  |
| Age, y | 55.0(47.0,62.5) | 64.0(58.0,71.0) | 64.0(58.0,70.0) | 63.0(57.0,68.0) | <0.001^**^ |
| DM duration, y | 8.0(3.0,15.0) | 11.0(7.0,18.0) | 10.0(4.0,17.0) | 12.0(7.0,19.0) | <0.001^**^ |
| BMI, kg/m^2^ | 25.8(23.4,28.3) | 25.8(23.5,27.9) | 25.6(23.4,28.6) | 25.0(22.9,27.6) | 0.187 |
| WHR | 0.94(0.9,1.0) | 0.94(0.9,1.0) | 0.93(0.9,1.0) | 0.93(0.9,1.0) | 0.08 |
| BP, mm Hg |  |  |  |  |  |
| Systolic | 135.0(123.0,151.0) | 141.5(127.0,153.0) | 138.0(127.0,153.) | 141.0(127.0,157.5) | 0.013^*^ |
| Diastolic | 78.0(70.0,86.0) | 76.0(70.0,83.0) | 76.0(69.0,83.0) | 77.0(69.0,84.0) | 0.374 |
| HbA1c, % | 8.4(7.1,9.7) | 8.1(7.0,9.5) | 7.8(6.7,9.4) | 7.8(6.7,9.2) | 0.003^**^ |
| Lipid profile, mmol/L |  |  |  |  |  |
| LDL-C | 2.71(2.1,3.3) | 2.64(2.0,3.4) | 2.74(2.1,3.4) | 2.84(2.2,3.6) | 0.063 |
| HDL-C | 1.21(1.0,1.4) | 1.23(1.1,1.4) | 1.28(1.1,1.5) | 1.33(1.1,1.6) | <0.001^**^ |
| TG | 1.47(1.0,2.3) | 1.36(1.0,2.0) | 1.42(1.0,2.1) | 1.46(1.0,2.3) | 0.499 |
| TC | 4.55(3.8,5.3) | 4.51(3.7,5.4) | 4.70(3.9,5.4) | 4.84(4.1,5.7) | 0.004^**^ |
| FFA | 0.43(0.3,0.6) | 0.43(0.3,0.6) | 0.41(0.3,0.6) | 0.45(0.3,0..6) | 0.537 |
| Statins | 105(62.5%) | 167(78%) | 174(78%) | 175(81.8%) | <0.001^**^ |

(Note: Data are presented as the median (interquartile range) for continuous variables or percentage for categorical variables. Abbreviations: BMI, body mass index; WHR, waist-to-hip ratio; DM, diabetes mellitus; BP, blood pressure; HbA1c, glycosylated hemoglobin; LDL-C, low-density lipoprotein cholesterol; HDL-C, high-density lipoprotein cholesterol; TG,triglyceride; TC, total cholesterol; FFA, free fatty acid.^a^Kruskal-Wallis H test or chi-square test. * Significant at p<0.05; ** Significant at p<0.01.)
