# Supplementary material for: Accelerated Ovarian Aging Among Type 2 Diabetes Patients and Its Association With Adverse Lipid Profile
Source: Front Endocrinol (Lausanne). 2022 Mar 30;13:780979. doi: 10.3389/fendo.2022.780979 (PMC9005646; doi:10.3389/fendo.2022.780979)
Supplement: Supplementary file 5 [file Table_4.docx]

**Supplemental table 4 Summary of clinical features of T2DM patients aged 51-60 years stratified by FSH quartiles**

| Characteristics | FSH quartiles | | | | P^a^ |
| --- | --- | --- | --- | --- | --- |
|  | Q1(n=242) | Q2(n=240) | Q3(n=242) | Q4(n=240) |  |
| Age, y | 54.0(47.0,58.3) | 65.0(60.0,71.0) | 64.0(58.0,70.0) | 63.0(58.0,71.0) | <0.001^**^ |
| DM duration, y | 8.0(3.0,13.0) | 10.0(6.0,18.0) | 10.0(5.0,17.0) | 12.0(7.0,19.0) | <0.001^**^ |
| BMI, kg/m^2^ | 26.00(23.6,28.7) | 25.89(24.0,28.3) | 25.45(23.4,28.1) | 25.02(22.8,27.2) | 0.002^**^ |
| WHR | 0.94(0.9,1.0) | 0.95(0.9,1.0) | 0.93(0.9,1.0) | 0.93(0.9,1.0) | <0.001^**^ |
| BP, mm Hg |  |  |  |  |  |
| Systolic | 134.0(123.0,152.0) | 141.0(129.0,154.0) | 139.0(127.8,153.3) | 141.0(126.3,156.0) | 0.017^*^ |
| Diastolic | 78.0(71.0,86.0) | 76.0(69.0,83.0) | 76.0(70.0,83.0) | 76.0(69.0,83.0) | 0.016^*^ |
| HbA1c, % | 8.46(7.3,9.7) | 8.40(7.0,9.7) | 7.80(6.8,9.3) | 7.60(6.6,8.9) | <0.001^**^ |
| Lipid profile, mmol/L |  |  |  |  |  |
| LDL-C | 2.66(2.1,3.3) | 2.78(2.1,3.4) | 2.70(2.1,3.3) | 2.80(2.3,3.6) | 0.074 |
| HDL-C | 1.18(1.0,1.4) | 1.23(1.1,1.4) | 1.28(1.1,1.5) | 1.36(1.2,1.6) | <0.001^**^ |
| TG | 1.46(1.0,2.4) | 1.44(1.0,2.1) | 1.38(1.0,2.1) | 1.37(1.0,2.0) | 0.596 |
| TC | 4.53(3.8,5.3) | 4.56(3.9,5.5) | 4.58(3.9,5.3) | 4.83(4.1,5.8) | 0.005^**^ |
| FFA | 0.44(0.3,0.6) | 4.56(3.9,5.5) | 4.58(3.9.5.3) | 1.37(1.0,2.0) | 0.844 |
| Statins | 94(57.7%) | 174(81.7%) | 174(76.7%) | 179(82.9%) | <0.001^**^ |

(Note: Data are presented as the median (interquartile range) for continuous variables or percentage for categorical variables. Abbreviations: BMI, body mass index; WHR, waist-to-hip ratio; DM, diabetes mellitus; BP, blood pressure; HbA1c, glycosylated hemoglobin; LDL-C, low-density lipoprotein cholesterol; HDL-C, high-density lipoprotein cholesterol; TG,triglyceride; TC, total cholesterol; FFA, free fatty acid.^a^Kruskal-Wallis H test or chi-square test. * Significant at p<0.05; ** Significant at p<0.01.)
